# Comparison of Liquid Chromatography Mass Spectrometry and Enzyme-Linked Immunosorbent Assay Methods to Measure Salivary Cotinine Levels in Ill Children

**DOI:** 10.3390/ijerph17041157

**Published:** 2020-02-12

**Authors:** E. Melinda Mahabee-Gittens, Matthew J. Mazzella, John T. Doucette, Ashley L. Merianos, Lara Stone, Chase A. Wullenweber, Stefanie A. Busgang, Georg E. Matt

**Affiliations:** 1 Division of Emergency Medicine, Cincinnati Children’s Hospital Medical Center, University of Cincinnati College of Medicine, Cincinnati, OH 45229, USA; lara.stone@cchmc.org (L.S.); chase.wullenweber@cchmc.org (C.A.W.); 2 Department of Environmental Medicine and Public Health, Icahn School of Medicine at Mount Sinai, New York, NY 10029, USA; matthew.mazzella@mssm.edu (M.J.M.); john.doucette@mssm.edu (J.T.D.); stefanie.busgang@mssm.edu (S.A.B.); 3 School of Human Services, University of Cincinnati, Cincinnati, OH 45221, USA; ashley.merianos@uc.edu; 4 Department of Psychology, San Diego State University, San Diego, CA 92123, USA; gmatt@sdsu.edu

**Keywords:** cotinine, ELISA, liquid chromatography, secondhand smoke exposure and children

## Abstract

**Objective**: Cotinine is the preferred biomarker to validate levels of tobacco smoke exposure (TSE) in children. Compared to enzyme-linked immunosorbent assay methods (ELISA) for quantifying cotinine in saliva, the use of liquid chromatography tandem mass spectrometry (LC-MS/MS) has higher sensitivity and specificity to measure very low levels of TSE. We sought to compare LC-MS/MS and ELISA measures of cotinine in saliva samples from children overall and the associations of these measures with demographics and TSE patterns. **Method**: Participants were nonsmoking children (N = 218; age mean (SD) = 6.1 (5.1) years) presenting to a pediatric emergency department. Saliva samples were analyzed for cotinine using both LC-MS/MS and ELISA. Limit of quantitation (LOQ) for LC-MS/MS and ELISA was 0.1 ng/mL and 0.15 ng/mL, respectively. **Results**: Intraclass correlations (ICC) across methods = 0.884 and was consistent in sex and age subgroups. The geometric mean (GeoM) of LC-MS/MS = 4.1 (range: < LOQ to 382 ng/mL; 3% < LOQ) which was lower (*p* < 0.0001) than the ELISA GeoM = 5.7 (range: < LOQ to 364 ng/mL; 5% < LOQ). Similar associations of cotinine concentrations with age ($\hat{\beta }$ < −0.10, *p* < 0.0001), demographic characteristics (e.g., income), and number of cigarettes smoked by caregiver ($\hat{\beta }$ > 0.07, *p* < 0.0001) were found regardless of cotinine detection method; however, cotinine associations with sex and race/ethnicity were only found to be significant in models using LC-MS/MS-derived cotinine. **Conclusions**: Utilizing LC-MS/MS-based cotinine, associations of cotinine with sex and race/ethnicity of child were revealed that were not detectable using ELISA-based cotinine, demonstrating the benefits of utilizing the more sensitive LC-MS/MS assay for cotinine measurement when detecting low levels of TSE in children.

## 1. Introduction

Biochemical verification is the gold standard used to validate levels of tobacco smoke exposure (TSE) in children and the tobacco use status of smokers in clinical trials [1,2,3,4,5,6]. Since pediatric TSE includes exposure from both secondhand smoke (SHS) and thirdhand smoke (THS), measurement needs to be reliable, highly sensitive and specific so that even low levels of THS exposure can be detected [1,7]. Nicotine is predominantly derived from combustible and noncombustible tobacco/nicotine products. Over 70% of nicotine is metabolized to cotinine by the liver enzyme CYP2A6 [8]. Cotinine is nicotine’s major proximate metabolite with a mean half-life of 16 h; some studies have reported higher median half-life levels of up to 28.3 h in infants and older children [1,9,10,11]. In combination with cotinine, the other nicotine metabolites are nicotine, trans-3’-hydroxy cotinine (3HC), cotinine glucuronide, nicotine glucuronide, and 3HC glucuronide, which in combination, estimates the approximate daily intake of nicotine [1,12]. Cotinine concentrations are relatively stable and can be detected in the blood, saliva, urine, hair, and nails of children who are exposed to SHS and THS [1]. Thus, cotinine is the most commonly reported measure to indicate children’s recent exposure to tobacco smoke. Since the concentration of cotinine in blood and saliva are highly correlated, saliva is often used to assess TSE in children since the collection is non-invasive, relatively simple, and well-tolerated [1,2].

The analytic methods most commonly used to assess cotinine are enzyme-linked immunosorbent assay (ELISA), liquid chromatography with triple quadruple tandem mass spectrometry (LC-MS/MS), and gas chromatography with mass spectrometry (GC-MS) [1]. Compared to ELISA, LC-MS/MS and GC-MS are considered to be state-of-the-art methods that are highly reliable and have excellent sensitivity and specificity to detect very low levels of TSE [1,13]. However, these highly specialized analyses require expensive equipment and large numbers of samples, and are lengthy and costly to conduct [1,12]. Since ELISA methods are more widely used and less cost-prohibitive, many studies that require biochemical validation have used ELISA assays to assess salivary cotinine levels in children [13,14,15,16,17,18]. It is unknown how ELISA assays compare to LC-MS/MS in measuring salivary cotinine. Additionally, since there is cross-reactivity of the ELISA method to cotinine, 3HC, and 3HC-glucuronide, levels of cotinine obtained via ELISA should be interpreted with caution [12]; however cross-reactivity is usually not considered in evaluating cotinine levels. To our knowledge, no studies have rigorously compared the results of salivary cotinine analyses using LC-MS/MS compared to ELISA. However, this comparison is needed to inform researchers about which assay method is suitable to accurately assess the primary outcomes of clinical trials and other studies, i.e., whether the goal is to assess low levels of SHS and THS or if the goal is to assess a broader, less precise measure of smoking status.

Further, it is recognized that child age and genetic variation in the frequency of CYP2A6 variant alleles among racial/ethnic groups [11,19] may result in differences in the rates and patterns of nicotine metabolism in children [1,20,21,22,23,24,25,26]. These differences are particularly pronounced in young children due to their increased exposure to tobacco smoke from active SHS or aged SHS (i.e., THS) due to close contact with caregivers who smoke, increased time spent indoors, increased contact with surfaces (e.g., floor, furniture), and high levels of hand-to-mouth behavior. This variability leads to differences in cotinine levels in children exposed to similar concentrations of nicotine (e.g., number of cigarettes smoked by their primary caregiver) from SHS or THS [2,8,27,28,29,30]. It is not known if these differences are observed and/or how they compare when measuring cotinine with LC-MS/MS and ELISA.

Thus, the primary objectives of this study were: to assess the cotinine levels of children who were exposed to varying levels of tobacco smoke in broad SHS and THS environments, and to compare the cotinine levels obtained using LC-MS/MS and ELISA. Secondary objectives were to determine how differences in cotinine levels differed by child demographics, TSE patterns, and home characteristics between the two methods. We hypothesized that LC-MS/MS would provide a superior measure of cotinine because: (1) better reliability means less error variance, and (2) lower limit of quantification (LOQ) indicates more valid quantitative measures for children with relatively low levels of exposure and less range restrictions. For these reasons, LC-MS/MS was expected to provide more power, fewer values <LOQ, and reduced range restriction attenuation bias.

## 2. Materials and Methods

### 2.1. Subjects and Biological Samples

Participants were 218 children of parental smokers enrolled in a randomized controlled trial (RCT) of a smoking cessation intervention who presented to the Pediatric Emergency Department (PED) or Urgent Care (UC) of a midwestern Children’s Hospital; details are described elsewhere [31]. Adult participants were parents or legal guardians of children 0–17 years of age who presented with a potentially TSE-related complaint (e.g., cough); enrollment occurred for a 28-month period beginning in April 2016. We included a convenience sample of participants from this study whose saliva samples were previously analyzed with ELISA techniques as part of the RCT. Leftover saliva samples from these participants were analyzed using LC-MS/MS. A total of 218 saliva samples were analyzed from children obtained at either the baseline PED/UC visit (T0; 203 participants) or during a 6-week home follow-up visit (T1; 15 participants); a single sample was tested per participant at either timepoint.

### 2.2. Measures

Parents completed electronic assessments that included demographics, TSE patterns, and type of home (e.g., multiunit housing). This study was approved by our hospital’s Institutional Review Board. Parental consent and child assent on children 11 years of age or older was obtained.

### 2.3. Chemical Analyses

Saliva was tested for cotinine and by the University of Minnesota using LC-MS/MS with isotope dilution and by Salimetrics LLC using ELISA techniques. The LOQ for the ELISA assay was 0.15 ng/mL [32]. For LC-MS/MS, salivary cotinine was analyzed as previously described [33] with the exception that the analyses were performed on a Luna C18 column. Briefly, the methods used were 96-well plate-based liquid chromatography-tandem mass spectrometry assays. Saliva cotinine was quantified by the area ratio of the analyte to the deuterated standard using specific MS/MS transitions. Calibration curves were established before each set of LC-MS/MS analyses. The same sample of deuterated analyte was used for constructing the calibration curve as that added to each sample as internal standard. Lower LOQ was calculated based on three times the noise measured in an extracted blank and was 0.1 ng/mL.

### 2.4. Statistical Analyses

Summary statistics of cotinine levels obtained with LC-MS/MS assays were compared to levels obtained with ELISA. Geometric means (GeoM) and standard deviations were calculated to account for the right skew of the data.

#### 2.4.1. Mixed Model

We used a one-way random effect mixed model comparing the final LC-MS/MS and ELISA-based cotinine measurements to calculate the intra-class correlations (ICCs) and to assess the variability attributed to the two methods. We also added the median relative percent difference (RPD) value to evaluate reproducibility in the presence of duplicate samples. Following the Environmental Protection Agency’s RPD guidelines, a median RPD less than 30% (or 0.3) indicates high quality of reproducibility [34]. For these mixed model analyses, only those samples with cotinine measurements above the LOQ for both methods were included (n = 203). For the LC-MS/MS and ELISA-based cotinine measurements, we had batch-to-batch replicate measurements for 20 and 55 participants, respectively, that were utilized to calculate the internal ICC and RPD for each method. For all mixed models, the cotinine concentrations were natural log (ln)-transformed to account for the right skew of the cotinine data. In follow-up analyses, models were stratified by sex and age group (0–6 and 7–17 years old).

#### 2.4.2. Paired Sample *t*-Test

A comparison of GeoMs for the LC-MS/MS and ELISA detected cotinine measures was performed by way of a paired sample t-test. As with the mixed models, we only included samples with both LC-MS/MS and ELISA cotinine measurements >LOQ (n = 203). All cotinine values were ln-transformed prior to analysis.

#### 2.4.3. Linear Regression Models

Associations of cotinine at T0 with explanatory variables were determined by way of linear regression models for each detection method. In order to ensure an accurate comparison of methods, only those samples with cotinine measured in both LC-MS/MS and ELISA at T0 were included in the models (n = 203); samples taken at the six-week timepoint (n = 15) were excluded from these analyses. Continuous explanatory variables included age of child (years), number of cigarettes smoked per day by primary caregiver, and number of cigarettes smoked per day around child by all household members. Categorical explanatory variables included child sex and race/ethnicity, type of home, and caregiver’s income. To account for the lower number of subjects in the Hispanic White and Black groups, race/ethnicity categories were collapsed to only include ‘non-Hispanic White’, ‘non-Hispanic Black’, and ‘other’. Similarly, categories for caregiver’s income were collapsed for income groups above $75,000. A total of six samples were dropped from the model due to missing explanatory variable data.

In contrast to the mixed model analyses, for the linear regression analyses, all values below LOQ were imputed as $\mathrm{LOQ}/\sqrt{2}$ using the detection method-specific LOQ values for both LC-MS/MS and ELISA-detected cotinine (3% and 5% of values imputed, respectively). Cotinine measurements were natural ln-transformed to account for the right skew of the data. An alpha of 0.05 was the criterion for statistical significance. All statistical analyses were conducted with SAS 9.4.

## 3. Results

### 3.1. Demographics and TSE Patterns

Participants (N = 218) were mostly non-Hispanic Black (55.1%) followed by non-Hispanic White (36.4%), and other race/ethnicity (8.4%); sex was evenly represented (51.4% female); mean age (SD) of child was 6.1 (5.1) years. A total of 37.3% of caregivers had an annual income of ≥ $15,000 and 47% lived in a single-family home. The mean (SD) number of cigarettes smoked by the primary caregiver and the total number of cigarettes smoked around the child was 9.8(6.0) and 9.1(16.7) cigarettes, respectively.

### 3.2. Cotinine Measurements and Comparisons of Analyses by LC-MS/MS and ELISA Distribution of Cotinine by LC-MS/MS Compared to ELISA

**Figure 1:**
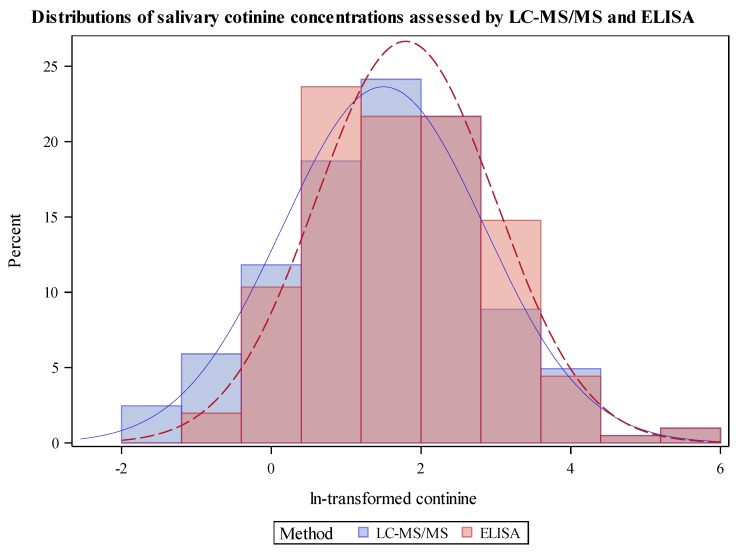
Distribution plot of ln-transformed cotinine in saliva (n = 203) detected by LC-MS/MS (blue) and ELISA (red).

**Figure 2:**
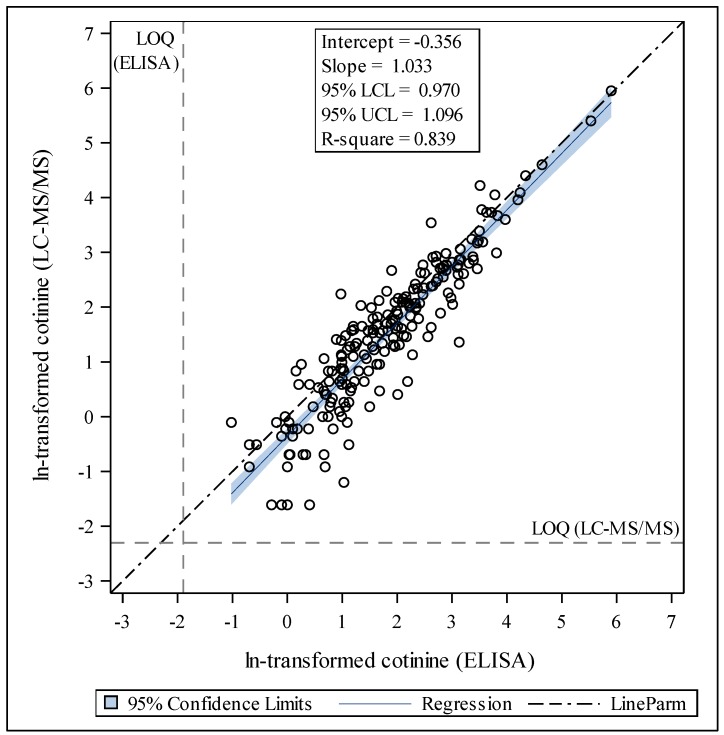
Scatter plot of LC-MS/MS vs. ELISA based ln-transformed cotinine measurements > LOQ (N = 203), with a linear regression line for the overall sample. Parameter estimates are provided in inset.

Cotinine was detected in 97% (n = 211) of saliva samples by LC-MS/MS and 95% (n = 208) of samples by ELISA. The LC-MS/MS assay showed higher sensitivity than the ELISA assay, with LOQs for each assay of 0.1 ng/mL and 0.15 ng/mL, respectively. Of the seven measurements < LOQ for LC-MS/MS, five were detectable by ELISA. Of the 10 measurements below LOQ by ELISA, eight were detected by LC-MS/MS. The GeoM for the LC-MS/MS assay was 4.1; median (Mdn) = 4.3 ng/mL; Q1 = 1.3 ng/mL; Q3 = 10.3 ng/mL; range <LOQ to 382 ng/mL. The GeoM for cotinine measured by ELISA was 5.7 ng/mL; Mdn = 5.1 ng/mL; Q1 = 2.2 ng/mL; Q3 = 12.2 ng/mL; range < LOQ to 364 ng/mL (Table 1). Based on samples with both LC-MS/MS and ELISA measured cotinine values > LOQ (n = 203), the GeoM for ELISA measured cotinine was significantly higher than LC-MS/MS measured cotinine (*p* < 0.0001). The distributions of the ln-transformed cotinine measurements were similar across method of detection (Figure 1). Scatter plots of LC-MS/MS-measured cotinine and ELISA-measured cotinine for samples with valid measurements in both methods showed that the measurements were largely in agreement (slope = 1.03, intercept = −0.36, R-square = 0.839; Figure 2). The negative intercept and slope >1 indicate that ELISA values tended to be higher than LC-MS/MS values, and this is more notable at lower levels. Stratification by age group and sex showed a similar trend that was more pronounced in in older subjects (7–17 years of age) and females.

Given the high range of cotinine values, we ran a sensitivity analysis excluding the top 5% values which was greater than 41.5 ng/mL for LC-MS/MS or greater than 41.2 ng/mL for ELISA. This reduced the sample size from 203 to 190. Of note, eleven of these thirteen children were under age five, thus, they were nonsmokers. There were no differences in the distributions or significance levels for Figure 1 or Figure 2 when we excluded these values.

### 3.3. Internal Consistency of LC-MS/MS and ELISA

Despite these areas of discrepancy, the calculated ICC indicates strong agreement of the LC-MS/MS and ELISA cotinine measurements with an overall ICC of 0.884 across methods and an ICC >0.82 and a median RPD <0.16 for all sex and age subgroups both across and within methods (Table 2).

### 3.4. Differences in Cotinine Results by Demographics, TSE, and Home Characteristics

Next, we assessed associations of cotinine in saliva at T0 with explanatory variables for samples assessed for cotinine by both LC-MS/MS and ELISA (n = 197) and compared the results (Table 3). Both models showed significant negative associations of age ($\hat{\beta }$ < −0.09, *p* < 0.0001) and positive associations of caregiver’s reported daily cigarette intake with cotinine ($\hat{\beta }$ > 0.07, *p* < 0.0001). Looking at the overall effect of categorical variables while controlling for covariates, there were significant effects of caregiver’s income with measured cotinine in saliva (*p* < 0.02), regardless of detection method. Plotting the residuals of a covariates-only model against income category indicated that the cotinine concentration broadly decreased as the categorical income level increased, regardless of detection method. Differences appeared between the models with LC-MS/MS-measured cotinine and ELISA-measured cotinine when looking at the effects of sex and race/ethnicity, where these effects were only significant in the model utilizing LC-MS/MS-measured cotinine. In the LC-MS/MS-based cotinine model, cotinine was shown to be lower in females than males and higher in non-Hispanic blacks than non-Hispanic whites, based on the covariate-adjusted plot. In separate models testing the possible interaction of race/ethnicity and age group, there were no indications of significant age by race/ethnicity interactions, regardless of cotinine detection method.

## 4. Discussion

To our knowledge, this is the first study conducted in a pediatric population to examine and compare salivary cotinine levels obtained with LC-MS/MS assays with levels obtained using ELISA assays. In this study of 218 children who were exposed to varying levels of tobacco smoke from SHS and THS in their environments, we observed that overall levels of cotinine were high.

The main objective of this study was to compare whether LC-MS/MS-based measures of salivary cotinine were comparable to the ELISA-based measures. Given the large differences in costs, turn-around time, and sensitivity and specificity between the two methods, investigators need to be able to weigh the potential trade-offs with each method so that they can assess which method may be more suitable for their planned research outcomes. For example, if investigators need to assess levels of TSE to broadly differentiate nonsmokers from smokers, then highly sensitive measures may not be needed. However, if cotinine levels are going to be used to differentiate those who are exposed to low levels of tobacco smoke, then more sensitive techniques are needed [1,23]. We observed good overall agreement with LC-MS/MS and ELISA in the relative ranking across the entire range of exposure. There were significant differences in the mean cotinine levels we observed with the GeoM for the LC-MS/MS assay of 4.1 compared to the ELISA GeoM of 5.7 ng/mL. These differences may be due to a number of reasons including the cross-reactivity of the ELISA assay with 3HC [12], but not LC-MS/MS, or because the ELISA calibration curves used with the ELISA assay were created based on studies conducted on adult participants [32]. It is possible that the ELISA calibration curves may need to be recalibrated for children.

The results show that both methods are internally highly reliable as demonstrated by ICCs > 0.98. The between method ICC, however, is substantially lower (0.82–0.90) and RPDs below 0.16, indicating that the two methods are subject to different sources of error that affect the observed cotinine levels. Using LC-MS/MS as the gold standard, ELISA cotinine measures may be affected by 10-18% of variance unrelated to actual interindividual differences in salivary cotinine levels. This is further illustrated by inconsistencies between the methods at the low exposure range. Of the seven measurements below LOQ for LC-MS/MS, five were detectable by ELISA. Of the 10 measurements below LOQ by ELISA, eight were detected with LC-MS/MS. Taking the LC-MS/MS measures as the gold standard, ELISA results have high rates of false positive results (five out of seven) at the low exposure range. Overall, these finding support the benefits of LC-MS/MS because of its superior specificity and invariance to cross-reactions and as the method of choice for populations with relatively low levels of exposure to tobacco smoke.

Our secondary objectives were to determine how differences in cotinine values based on child demographics, TSE patterns, and housing types compared between the two methods. Overall, our results were consistent with prior research in that we observed higher cotinine levels using both methods in children who were: younger [35,36,37]; had lower household incomes [36,38,39]; and had higher numbers of cigarettes smoked by their primary caregiver or around them [24,39]. We did, however, observe differences in cotinine associations with sex and race/ethnicity that were only significant in models using LC-MS/MS-derived cotinine levels. We found that cotinine levels were lower in females and higher in non-Hispanic blacks than non-Hispanic whites. Other studies have not reported differences in cotinine by gender [24,35,37,40] and it is unclear why this is the case. However, similar to our findings, several other studies have observed differences by race/ethnicity with higher cotinine levels seen in non-Hispanic blacks. These differences may be due to differences in nicotine metabolism due to genetic variation in the CYP2A6 enzyme [8,11,19]. It appears that a relatively high proportion of African American smokers (15% compared to 1% in Caucasian smokers) have a particular variant in their metabolic pathway that contributes to significantly higher cotinine levels per cigarette [41]. However, it is not clear how these racial/ethnic differences in nicotine metabolism translate to TSE and cotinine levels in children.

Our findings are not without limitations. Our sample consisted of ill children recruited from the PED/UC. While we chose to collect and analyze saliva in our population due to the ease of collection and tolerability compared to collection of plasma or urine, especially in young children, we acknowledge that there are limitations to assessing levels of TSE using saliva. Salivary cotinine concentrations can be affected by age, sex, race, oral pH, type of diet, dehydration, or drug treatment [1]. All of these factors were highly variable in our population of ill children. Nevertheless, other studies have found salivary cotinine levels to be comparable to plasma cotinine levels in adolescent and adult smokers [42,43], although levels are approximately 10% to 40% higher [1,42,43,44]. Additionally, our saliva collection method (i.e., the use of cotton swabs as recommended by Salimetrics [32]) may have resulted in differential salivary flow rate which also could have affected cotinine levels [42]. Further, the timing of saliva collection may have lowered cotinine levels if saliva was obtained after the child had been in the PED/UC for a while, since cotinine has a mean half-life of 16–18 h [8]. Since we enrolled a convenience sample of participants, our results cannot be generalized to different racial/ethnic groups or other sociodemographic subgroups. Moreover, since our population was highly exposed to tobacco smoke, using ELISA assays on children who had lower levels of exposure or who were predominantly exposed to THS may have resulted in more results below the LOQ. Finally, since cotinine levels obtained using LC-MS/MS on urine samples yield lower LOQs, urine samples may be a better measure of TSE when there are low levels of exposure [1] and studies are needed to compare these salivary cotinine results with urinary cotinine results obtained with both methods.

## 5. Conclusions

In conclusion, our results indicate that ELISA is a cost-effective alternative to LC-MS/MS for detecting TSE to classify children into highly exposed versus not exposed. However, LC-MS/MS is a superior method with which to measure cotinine in children with lower levels of TSE. This is evident given our results that the associations of cotinine with sex and race/ethnicity were detected only when cotinine was quantified by LC-MS/MS and because we had fewer values that were below the LOQ with LC-MS/MS. These results demonstrate the benefits of utilizing the more sensitive LC-MS/MS assay for cotinine measurement in children when detecting TSE, especially when low levels of exposure to nicotine via THS or SHS are to be measured. Future research should include validation and calibration studies of ELISA-based cotinine methods for pediatric populations and studies of LC-MS/MS-based measures with a broader range of TSE to better understand the reliability and validity of measuring TSE in age and racially/ethnically diverse populations.

## Figures and Tables

**Table 1:** Percent detected, geometric mean (GeoM), geometric standard deviation, and distribution of cotinine in children’s saliva (N = 218) as detected by liquid chromatography tandem mass spectrometry (LC-MS/MS) and enzyme-linked immunosorbent assay (ELISA).

Analyte	Method	Total Samples Analyzed	Total Samples Detected	Percent Detected	LOQ	GeoM	Geometric SD	Min	P25	P50	P75	P95	Max
Cotinine (ng/mL)	LC-MS/MS	218	211	97%	0.10	4.1	4.1	<LOQ	1.3	4.3	10.3	41.5	382
	ELISA	218	208	95%	0.15	5.7	3.5	<LOQ	2.2	5.1	12.2	41.2	364

Min = Minimum; P25 = 25th percentile; P50 = 50th percentile; P75 = 75th percentile; P95 = 95th percentile; Max = Maximum. Abbreviations: LOQ, limit of quantitation.

**Table 2:** Intra-class correlation coefficient (ICC) and median relative percent difference (RPD) of the LC-MS/MS and ELISA based cotinine measurements overall and by sex and age of child subgroups. Across method comparison utilized ln-transformed final best values. Internal method ICC and median RPD calculated using ln-transformed values from replicate samples, which were available from a subset of subjects for LC-MS/MS (n = 20) and ELISA (n = 55).

			Internal ELISA (N = 55)		Internal LC-MS/MS (N = 20)	
		Across methods (N = 203)	ICC	Median RPD *	ICC	Median RPD
**Overall**		0.884	0.993	0.113	0.991	0.061
**Age**	**0–6 yrs (n = 122)**	0.899	0.992	0.118	0.996	0.057
	**7–17 yrs (n = 81)**	0.824	0.994	0.099	0.983	0.073
**Sex**	**Male (n = 100)**	0.890	0.993	0.087	0.995	0.056
	**Female (n = 103)**	0.869	0.993	0.156	0.985	0.073

* For the RPD, we use the formula: RPD = (abs(X1 - X2))/$\bar{X}$ where X1 is the concentration of replicate 1 and X2 is the concentration of replicate 2 and $\bar{X}$ is the mean of the two concentrations.

**Table 3:** Results of linear regression model of ln-transformed cotinine baseline measurements testing for associations with predictor variables by cotinine measurement method (LC-MS/MS and ELISA; N = 197). Parameter estimates, 95% confidence intervals, and p values of overall effect provided for continuous and categorical explanatory variables. Categorical explanatory variables have parameter estimates provided for each category (italicized) in relation to reference category.

		LC-MS/MS Based Cotinine				ELISA Based Cotinine			
Parameter	% of Cohort	$\hat{\beta }$	95% LCL	95% UCL	*p*-Value	$\hat{\beta }$	95% LCL	95% UCL	*p*-Value
**Age of child**	N/A	−0.11	−0.15	−0.08	< 0.0001	−0.09	−0.13	−0.06	< 0.0001
**# cigarettes/day smoked-caregiver**	N/A	0.09	0.05	0.12	< 0.0001	0.07	0.04	0.11	< 0.0001
**# cigarettes/day smoked around child**	N/A	0.01	−0.00	0.02	0.17	0.01	−0.01	0.02	0.34
**Sex of child**									
*Female*	51.3%	Ref	Ref	Ref	Ref	Ref	Ref	Ref	Ref
*Male*	48.7%	0.38	0.02	0.75	0.04	0.30	−0.06	0.65	0.10
**Race**/**ethnicity**									
*Non-Hispanic White*	36.6%	Ref	Ref	Ref	Ref	Ref	Ref	Ref	Ref
*Non-Hispanic Black*	54.3%	0.52	0.10	0.95	0.04	0.38	−0.03	0.80	0.16
*Other*	9.1%	0.07	−0.61	0.75	0.04	0.06	−0.61	0.73	0.16
**Caregiver’s Annual Income**									
*Less than $5,000*	35.5%	Ref	Ref	Ref	Ref	Ref	Ref	Ref	Ref
*$5,001 to $15,000*	26.9%	0.10	−0.35	0.55	0.01	0.04	−0.40	0.47	0.02
*$15,001 to $30,000*	21.3%	−0.44	−0.93	0.05	0.01	−0.49	−0.97	−0.01	0.02
*$30,001 to $50,000*	9.1%	−1.07	−1.74	−0.41	0.01	−0.91	−1.56	−0.27	0.02
*$50,001 to $75,000*	5.6%	−0.48	−1.29	0.34	0.01	−0.43	−1.23	0.36	0.02
*More than $75,000*	1.5%	−0.98	−2.50	0.55	0.01	−1.55	−3.04	−0.06	0.02
**Type of home**									
*Single family house*	48.2%	Ref	Ref	Ref	Ref	Ref	Ref	Ref	Ref
*Multi-family house*	20.3%	0.49	0.02	0.96	0.06	0.22	−0.24	0.68	0.19
*Apartment*	30.0%	0.53	0.10	0.97	0.06	0.45	0.02	0.87	0.19
*Other*	1.5%	0.66	−0.82	2.14	0.06	−0.37	−1.81	1.08	0.19

Ref-reference category; LCL-lower confidence level; UCL-upper confidence level.
